# Epizootiologic Parameters for Plague in Kazakhstan

**DOI:** 10.3201/eid1202.050651

**Published:** 2006-02

**Authors:** Michael Begon, Nikolay Klassovskiy, Vladimir Ageyev, Bakhtiar Suleimenov, Bakhyt Atshabar, Malcolm Bennett

**Affiliations:** * University of Liverpool, Liverpool, United Kingdom;; † Kazakh Scientific Centre for Quarantine and Zoonotic Diseases, Almaty, Kazakhstan

**Keywords:** Plague, Yersinia pestis, zoonosis, epizootiology, gerbil, Rhombomys, reservoir, research

## Abstract

Reliable estimates are lacking of key epizootiologic parameters for plague caused by *Yersinia pestis* infection in its natural reservoirs. We report results of a 3-year longitudinal study of plague dynamics in populations of a maintenance host, the great gerbil (*Rhombomys opimus*), in 2 populations in Kazakhstan. Serologic results suggest a mid-summer peak in the abundance of infectious hosts and possible transmission from the reservoir to humans. Decrease in antibody titer to an undetectable level showed no seasonal pattern. Our findings did not support the use of the nitroblue-tetrazolium test characterization of plague-infected hosts. *Y. pestis* infection reduced survival of otherwise asymptomatic hosts.

## Abstract 2

Infection reduces survival of otherwise asymptomatic maintenance hosts in a natural reservoir population.

Plague, which is caused by infection with *Yersinia pestis* and usually transmitted by fleas, is enzootic in many parts of Asia, Africa, and North and South America. Its natural reservoirs are usually rodent species outside the peridomestic environment, but peridomestic rodents may act as important liaison hosts between the sylvatic reservoir and humans (*1*). In addition to its widely acknowledged historic importance, plague can now be classified as an emerging disease. Land-use changes in many parts of the world are increasing the probability of interaction between sylvatic rodents and humans, and between sylvatic and peridomestic rodents (*1*). Also, throughout much of central Asia, support for surveillance programs that previously screened for and treated plague outbreaks in the rodent reservoirs is now being withdrawn. Furthermore, in the United States in particular, acknowledgement of the risk of plague in humans is increasing, in part because of human cases beyond normal foci (*2*) and in part because of the threat of bioterrorism (*3*).

However, many gaps remain in understanding the dynamics and natural history of plague in its natural reservoirs (*4**,**5*). Thus, problems arise when attempts are made to model plague-flea-rodent systems, which require parameter estimates for important processes (6,7, S. Park et al., unpub. data), or when these models generate parameter estimates that cannot be checked against independent values more directly derived from field data (S. Park et al., unpub. data). Moreover, work on the epizootiology of plague in different continents has been conducted largely independently, with researchers using different methods (*4*). Under these circumstances, contrasting paradigms accompanied by mutual skepticism may be produced. As Gage and Kosoy (*5*) have commented, "The importance of research on the natural history of plague can hardly be questioned, as it has provided critical information for the development of effective plague prevention and control techniques, but often contradictory results point out the need for studies designed to test specific hypotheses."

Our study is a first attempt to link these independent traditions. We report the results of a study carried out in the plague focus in eastern Kazakhstan, where other data have also been gathered as part of the surveillance system of the former Soviet Union, a function now carried out by the national government. The main reservoir host is the great gerbil, *Rhombomys opimus*, as it is throughout large tracts of central Asia (*8**–**10*). This animal is an enzootic or maintenance host (*5*) because, although plague is frequently reported in this species, often with a high prevalence (especially serologically), mass deaths of the host are not observed.

Great gerbils live in family groups, typically a single male, 1 or a few females, and their immature offspring, that inhabit and defend discrete, permanent burrow systems (*11*). The number, position, and size of the burrow systems generally do not change over time, but the proportion occupied by family groups (occupancy) may fluctuate dramatically (*12*). The vectors of plague are primarily fleas in the genus *Xenopsylla* that inhabit the burrow systems of the great gerbil (*13*).

The data were obtained from 2 nearby but independent sites monitored in parallel. Samples were taken repeatedly from the same sites and, when possible, from the same (marked) individual gerbils. Evidence of infection in individual animals was sought both directly, by isolation of *Y. pestis* (though isolates were rare), and by serologic analysis.

The following questions were addressed. First, what accounts for variation in the probability of recapture, and thus of survival, in great gerbils? We attempted to determine whether plague has a detectable effect on survival of asymptomatic gerbils and whether seasonal variations in survival exist to make accurate assumptions in analyses of long-term (e.g., biannual) datasets on infection (S. Park et al., unpub. data).

Second, what accounts for variations in seroprevalence (the proportion of the population seropositive for antibody to plague), variations in the antibody titer of seropositive animals, and variations in the loss of detectable levels of antibody by these animals? These questions address patterns of infection: e.g., is infection acquired during particular seasons and by particular age classes of host? Understanding patterns in the acquisition and loss of antibody may be essential in interpreting long-term data, which are often based on serologic results (S. Park et al., unpub. data).

Third, what accounts for variations in nitroblue-tetrazolium (NBT) test values? The NBT test (*14*) measures the percentage of actively phagocytosing (positively stained) neutrophils in blood smears. Some Russian plague literature relies on it to provide supplementary (indeed crucial) information on infection status in wild rodents, e.g., whether infection is at an early or late stage (*15*). However, the test has not been adopted by plague workers elsewhere, and its value is open to question. We attempted to resolve the issue of its usefulness.

## Methods

The 2 study sites were in Kizil-Dzar (site 1; 500 m × 600 m) and Shagildi (site 2; 500 m × 500 m), located ≈40 km apart in an area southeast of Lake Balkhash in eastern Kazakhstan. This area is a desert with sandy soil and sparse vegetation, primarily black saxaul (*Haloxylon aphyllum*), white saxaul (*H. persicum*), a number of grasses (especially *Anabasis ramosissima* and *Ceratocarpus turkestanicus*), and sandy sedge (*Carex physodes*).

All gerbil colonies at the sites were mapped, although not all were occupied at any time. Site 1 had 76 colonies and site 2 had 87 colonies. The populations at the 2 sites were sampled every month from January 2002 to July 2004, except when problems of access occurred, mostly due to inclement weather, especially during the winter of 2003–2004. Samples were obtained 25 times from each population at the 2 sites. Unbaited wooden traps were placed at entrances to occupied burrows (which showed signs of recently disturbed sand) and checked twice a day over a 3- to 4-day period. Traps were not left in position overnight. Gerbil abundance, as estimated by the proportion of burrows occupied (*12*), increased to a peak each mid-summer. It was somewhat higher and more constant from year to year at site 1 than at site 2, and was lowest at site 2 in 2003. Captured animals were identified by sex, weighed, and classified as juveniles, subadults, or adults on the basis of size, weight, and coat color.

Fleas on the captured gerbils were collected with fine forceps. A blood sample was obtained from the tip of the gerbil's tail, and, if captured for the first time, it was injected subcutaneously with a microchip transponder so that its unique identity (9-number code) could be determined on recapture. Blood samples were analyzed for infection with *Y. pestis* by culturing on Hottinger's agar containing 1% hemolyzed sheep erythrocytes. Colonies obtained were identified as *Y. pestis* by colony morphologic features, sensitivity to diagnostic plague bacteriophage, and the presence of F1 capsular antigen. Blood samples were also tested serologically for F1 antigen by undirected hemagglutination and confirmed by hemagglutination inhibition with F1 antigen (*16*). A blood sample was used for the NBT test (*14*) in which 400 neutrophils from a blood smear were observed under 400× magnification to determine the proportion that were positively stained and showed evidence of active involvement in phagocytosis. Fleas were also monitored for active bacteria; these results will be reported in a subsequent publication.

A field-scale experiment was conducted in which half of the burrows at site 2 were treated with insecticide between November 2003 and the end of the observation period to rid them of fleas. (Detailed results will be described in a subsequent report.) Such treatment was not included as an explanatory variable, but if it affected any of the response variables, this should emerge as a significant site × time interaction.

Five response variables were analyzed by using generalized linear modeling. Recapture, defined as whether an animal was captured subsequently after release, was analyzed with a logit link and binomial errors. This is discussed as a proxy for survival, which clearly codetermines the probability of recapture along with emigration and ease of being caught. Seropositivity, defined as whether an animal exhibited demonstrable levels of antibody, was also analyzed with a logit link and binomial errors. Antibody titer was analyzed with an identity link and Gaussian errors. Loss of seropositivity, defined as whether an animal that was seropositive on release was still seropositive on subsequent recapture, was analyzed by using a logit link and binomial errors. NBT value was analyzed with logit link and binomial errors. For antibody titers, results of 2 laboratory tests showed a strong correlation; thus, results of statistical analyses were always effectively identical. For simplicity, antibody refers to the results of a passive hemagglutination test.

The following explanatory variables were examined initially (when they were not response variables): sex, site, maturity (adult, subadult, or juvenile), recapture, seropositivity, antibody titer, NBT value, year of capture, and month of capture. If a clear pattern was apparent in the full model in the signs of coefficients for different months, then to simplify and detect seasonal patterns, we tested a model with season based on the coefficients against the model that included all months.

Model selection was based on Akaike information criterion (AIC), which attempts to find the simplest model that adequately explains data by trading-off reductions in residual deviance against the number of parameters used in a model (see [17] for a review of model selection approaches). Models with a difference in AIC (ΔAIC) <2 can be considered indistinguishable in their explanatory power (*18*). For reasons of practicality in both analysis and interpretation, only 2-way interactions between explanatory variables were examined.

## Results

For recapture (as a proxy for survival), initial exploration of competing models of the data (n = 1,360) showed a clear pattern in the sign of the coefficients of different months, which were positive from July through October but negative or zero from November through June, with the exception of April (coefficient = 0.068, standard error [SE] = 0.56). Therefore, a model with July–October classified as summer and November–June as winter (season) subsequently replaced month. The optimal model (AIC = 1,458.5) included season, maturity, year, and seropositivity, along with interactions between season and year and between age and year. Coefficients and their significances are shown in Table 1. The closest model (AIC = 1,460.1, ΔAIC ≈2) excluded seropositivity. Thus, inconclusive evidence supported the conclusion that seropositive animals are less likely to survive than seronegative animals. The probability of recapture was also much lower in the third year of the study and significantly lower in the second year than in the first year. This finding was at least in part an inevitable consequence of many animals released in the third year and even the second year still being alive (but not yet recaptured) when the study terminated. This effect was also responsible for the important interactions in the model. Thus, overall, recapture rates were lower for winter releases but were also low for the summer of the third year, at the end of the study. Similarly, the overall trend was for recapture rates to be lower for animals released as juveniles, but this was marked only in the first year, whereas rates were lowest for adults released in the third year.

**Table 1:** Estimates of coefficients, standard errors, and significance based on z tests for optimal generalized linear model for whether animals were recaptured after release

Comparator	Effect	Estimate	Standard error	z value	p(>|z|) value*
	Intercept	–0.098	0.17	–0.59	0.56
Summer	Winter	–1.12	0.20	–5.48	4.3 × 10^-8^
Adult	Subadult	0.09	0.23	0.41	0.69
Adult	Juvenile	–0.50	0.32	–1.57	0.12
Year 1	Year 2	–1.23	0.25	–5.00	5.5 × 10^-7^
Year 1	Year 3	–4.85	1.49	–3.25	0.0011
Antibody negative	Positive	–0.30	0.16	–1.90	0.058
Summer × year 1	Winter × year 2	0.85	0.34	–2.47	0.014
Summer × year 1	Winter × year 3	4.12	1.48	2.80	0.0051
Adult × year 1	Subadult × year 2	0.99	0.35	2.85	0.0044
Adult × year 1	Subadult × year 3	1.32	0.48	2.77	0.0056
Adult × year 1	Juvenile × year 2	2.43	1.55	1.57	0.12
Adult × year 1	Juvenile × year 3	3.70	1.21	3.05	0.0023

*Probability of exceeding the z value by chance alone.

For seropositivity (whether animals had detectable levels of plague antibody; n = 1,287), the optimal model (AIC = 1,287.6; Table 2) included maturity, year, site, NBT value, and whether an animal was subsequently recaptured, as well as a large number of interactions. The closest model (AIC = 1,287.7), and the only model to come near the optimal model, also included an interaction between site and whether an animal was recaptured. Subadults were significantly less likely to be seropositive than adults, and juveniles were even less likely, especially in the later years of the study. Seropositivity was significantly associated with high NBT values, especially in subadults and juveniles, and particularly at site 2. A low likelihood of seropositivity was found at site 2 in the third year of the study. Finally, animals that were subsequently recaptured were less likely to be seropositive.

**Table 2:** Estimates of coefficients, standard errors, and significance based on z tests for optimal generalized linear model for whether animals were seropositive for antibody to Yersinia pestis*

Comparator	Effect	Estimate	Standard error	z value	p(>|z|) value†
	Intercept	–1.00	0.19	–5.19	2.07 × 10^–7^
Adult	Subadult	–0.73	0.31	–2.37	0.018
Adult	Juvenile	–1.94	0.61	–3.19	0.0015
Year 1	Year 2	0.15	0.27	0.55	0.58
Year 1	Year 3	0.534	0.53	1.00	0.32
	NBT	2.54	0.88	2.88	0.0040
Site 1	Site 2	–0.36	0.26	–1.39	0.16
Not recaptured?	Recaptured	–0.35	0.17	–2.08	0.037
Adult × year 1	Subadult × year 2	–1.76	0.50	–3.49	0.00049
Adult × year 1	Juvenile × year 2	–0.11	0.66	–0.16	0.87
Adult × year 1	Subadult × year 3	–2.91	1.09	–2.68	0.0073
Adult × year 1	Juvenile × year 3	–11.90	401.1	–0.03	0.98
Adult × NBT	Subadult × NBT	7.79	2.25	3.47	0.00053
Adult × NBT	Juvenile × NBT	9.47	3.89	2.43	0.015
Year1 × NBT	Year 2 × NBT	–3.60	1.49	–2.41	0.016
Year1 × NBT	Year 3 × NBT	–7.59	3.07	–2.47	0.013
Site1 × year1	Site 2 × year 2	0.26	0.33	0.80	0.42
Site1 × year1	Site 2 × year 3	–2.97	1.14	–2.60	0.0094
Site1 × NBT	Site 2 × NBT	6.42	1.68	3.81	0.00014

*NBT, nitroblue-tetrazolium. †Probability of exceeding the z value by chance alone.

Among seropositive animals, initial exploration of competing models for antibody titer (n = 342) again showed a significant effect of month and a clear pattern in the sign of the coefficients, but in this case September and October had positive coefficients, whereas all others were negative or zero. A model with September and October classified as "autumn" and other months as "other" was far superior (ΔAIC = 14.4), and autumn therefore replaced month subsequently. The model with the lowest AIC (AIC = 1,524.2; Table 3) included autumn, maturity, and whether animals were recaptured subsequently, with no interactions. However, the closest model (AIC = 1,525.0, ΔAIC = 0.8) excluded maturity, which indicated only weak support for a maturity effect. However, antibody titers were significantly more likely to be higher in September and October. They also tended to be higher in animals that were subsequently recaptured.

**Table 3:** Estimates of coefficients, standard errors, and significance based on t tests for optimal generalized linear model for variations in antibody titer among seropositive animals

Comparator	Effect	Estimate	Standard error	t value	p(>|t|) value*
	Intercept	8.13	0.38	21.66	<2 × 10^-16^
Autumn	Other	–1.81	0.36	–5.07	6.74 × 10^-7^
Adult	Subadult	0.47	0.34	1.37	0.17
Adult	Juvenile	–0.76	0.52	–1.46	0.15
Not recaptured?	Recaptured	0.47	0.26	1.78	0.077

*Probability of exceeding the t value by chance alone.

Among seropositive animals, models that explore factors affecting loss of seropositivity must be interpreted with particular caution because the requirement that animals be recaptured after a seropositive result led to the lowest sample size (n = 81). The optimal model (AIC = 56.3; Table 4) included only antibody titer on release, lower values of which made loss of seropositivity more likely. The only model to come close to this (AIC = 56.9, DAIC = 0.6) also included a weak suggestion that a longer gap between release and recapture made loss more likely. Attempts to account for variations in NBT values (n = 1,335) failed to uncover any factors that improved on an intercept-only model; the minimum DAIC for a model that also included seropositivity was 2.76.

**Table 4:** Estimates of coefficients, standard errors, and significance based on z tests for optimal generalized linear model for whether seropositive animals that were released were seronegative on subsequent recapture

Effect	Estimate	Standard error	z value	pr(>|z|) value*
Intercept	1.01	0.70	1.44	0.15
Antibody titer	–0.64	0.18	–3.48	0.00051

*Probability of exceeding the z value by chance alone.

## Discussion

The control of plague, in particular the control of the risk of it spreading from its wildlife hosts into peridomestic animals and humans, depends on understanding the dynamics and natural history of plague in those wildlife hosts. This study investigated 3 aspects of plague in its natural hosts in one of the world's major plague foci: the effect of infection on host survival, the dynamics of the antibody response to infection, and the specificity of the NBT test, which has been used as a measure of rodent infection status in previous studies.

From the point of view of host dynamics, an important finding from these analyses was that seropositive animals were less likely than seronegative animals to be recaptured. (This was mirrored in the analysis of seropositivity: animals subsequently recaptured were less likely to be seropositive.) This situation could arise through any combination of the following features: increased death, increased emigration, and greater difficulty in being caught among seropositive animals. However, since greater difficulty in being caught implies abnormal behavior, and rodents leaving their natal territory often have lower survival rates (*19*), these results suggest that death is increased among seropositive animals. Furthermore, the presence of antibody may indicate present or recent infection, or an infection that had been cleared many months previously. Thus, any effect of plague infection on host survival can only be conservatively estimated by an effect of seropositivity. These results further suggest that the effect of plague infection on host survival may be substantial. Plague-induced death in susceptible hosts, such as prairie dogs (*Cynomys* spp.) in the United States, which have extensive "die-backs," is well established (*20*). More subtle effects on asymptomatic animals of resistant species have not been reported for plague (*4*), but they have the potential to affect the dynamics of host populations (*21*). Variations in great gerbil abundance are, in turn, critical in determining the public health risk of plague (*12*), and future development of models designed to predict outbreaks and help manage public health risk (*8**,**12*) should take such effects into account.

In spite of the influence of inevitably lower recapture rates at the end of the study period (not associated with any decrease in abundance), recapture rates tended to be lower in juveniles, a common finding in rodent populations (*21*), and likely reflected both higher emigration rates and lower survival among young animals yet to establish themselves in the population. The recapture rate also showed seasonal variation; it was higher for animals released from July through October, a period of peak abundance (burrow occupancy) when the population is dominated numerically by adults that will delay breeding until spring of the following year.

Since many factors interacted to determine recapture rates, determining typical recapture rates is not possible. However, the probability of being recaptured for a seropositive animal was ≈84% of that for an equivalent seronegative animal (e.g., for an adult in summer in year 1, ≈40% compared with 48%); the probability of being recaptured if released in winter was ≈50% that if released in summer (for a seronegative adult in year 1, 23% compared with 48%). The probability for juveniles was ≈70% of that for adults and subadults (for seronegative animals in summer in year 1, 35% compared with 50%).

The analysis of seropositivity indicated that adults (who had a longer time to acquire infection) were most likely to be seropositive and that juveniles (who had the least time) were least likely. For animals at site 1 in year 1 who were not subsequently recaptured, the probabilities of being seropositive were 27%, for adults, 15% for subadults, and 5% for juveniles. Furthermore, antibody titers were highest in September and October, soon after the peak period for the acquisition of new infections, after an influx of newborn susceptible animals into the population in mid-summer. For adults who were not subsequently recaptured, average titers were ≈1:2,560 (denoted as 8, the intercept value, in Table 3) in September and October, compared with titers ≈1:640 during the rest of the year. High titers in subadults (Table 3) are likely to reflect the high proportion of recent infections in this age group, whereas low titers in juveniles may reflect samples taken in the very earliest stages of infection, when titers are still increasing. Higher titers in animals recaptured may also reflect a pattern in which the oldest animals were both less likely to survive and more likely to have antibody titers that had decreased to low levels. The lower seroprevalence at site 2 in the third year of the study suggests that treatment of half of this site for fleas in autumn of the second year was successful in reducing plague transmission. Although no seasonal pattern in seropositivity was found, the serologic results suggest a clear mid-summer peak in the abundance of infectious hosts and a possible public health risk because of the sylvatic reservoir. Such risk will also depend on variations in reservoir-human contact rates associated with changes in host and human behavior, which would ideally be included in any risk-management model.

In spite of the inevitably low sample size resulting from stringent criteria for inclusion, the level of antibody was likely to decrease to undetectable levels in animals released with a low titer, particularly if the period of antibody decrease from release to recapture was long. However, no detectable seasonal or age-related effects were detected. Such negative results are important because all existing time series on the dynamics of plague, great gerbils, and fleas in central Asia comprise data collected biannually. Analyses of these data (*7*, S. Park et al., unpub. data) assume a seasonal structure in values of key parameters (loss of antibody, survival), and suggest such a structure as part of a statistical model deemed most likely to account for the biannual time series. Thus, results such as those reported here, which reflect direct observations at intervals appropriate to the processes being observed, can test the validity of assumptions and seek to confirm (or contradict) statistical models. The observation that survival is lower in winter does not confirm the assumption (S. Park et al., unpub. data) that winter and summer rates are the same, while the absence of a seasonal pattern of antibody loss does not confirm the conclusion from modeling that loss appears to be greater over winter.

Values in the NBT test, which may be an indication of the proportion of neutrophils actively involved in phagocytosis, especially during acute bacterial infections (*14*), have been used in some studies to classify animals positive for antibody to *Y. pestis* into different subgroups. The profile of values within a population would be indicative of different phases of the progression of a plague epizootic and of the consequent risk to humans (*15*). In the present analyses, NBT values were higher in seropositive animals, but none of the factors measured, including seropositivity, accounted for a significant amount of the variation in NBT values. This finding suggests that although activity of neutrophils may respond to *Y. pestis* infection, this activity is also equally or more responsive to other factors not measured here (almost certainly including other infections). Thus, the usefulness of NBT values in determining public health risk must be questioned.
